# Synthesis and Characterization of pH-Responsive PEG-Poly(β-Amino Ester) Block Copolymer Micelles as Drug Carriers to Eliminate Cancer Stem Cells

**DOI:** 10.3390/pharmaceutics12020111

**Published:** 2020-01-30

**Authors:** Weinan Li, Jialin Sun, Xiaoyu Zhang, Li Jia, Mingxi Qiao, Xiuli Zhao, Haiyang Hu, Dawei Chen, Yanhong Wang

**Affiliations:** 1 School of Pharmacy, Shenyang Pharmaceutical University, Shenyang 110016, China; tyler2046@163.com (W.L.);; 2 School of Pharmacy, Heilongjiang University of Chinese Medicine, Harbin 150040, China; ZXY1773971575@163.com; 3 Key Laboratory of Forest Plant Ecology, Northeast Forestry University, Ministry of Education, Harbin 150040, China; klp15sjl@nefu.edu.com; 4 Department of Pharmacy, Heze Medical College, Heze 274000, China; jiali0978@163.com

**Keywords:** poly(β-amino ester), polymeric micelles, pH-responsive, cancer stem cells, thioridazine

## Abstract

PEG-poly(β-amino ester) (PEG-PBAE), which is an effective pH-responsive copolymer, was mainly synthesized by Michael step polymerization. Thioridazine (Thz), which was reported to selectively eliminate cancer stem cells (CSCs), was loaded into PEG-PBAE micelles (PPM) prepared by self-assembly at low concentrations. The critical micelle concentrations (CMC) of PPM in water were 2.49 μg/mL. The pH-responsive PBAE segment was soluble due to protonated tertiary amine groups when the pH decreased below pH 6.8, but it was insoluble at pH 7.4. The Thz-loaded PEG-PBAE micelle (Thz/PPM) exhibited a spherical shape, and the drug loading was 15.5%. In vitro release of Thz/PPM showed that this pH-sensitivity triggered the rapid release of encapsulated Thz in a weakly acidic environment. The in vitro cytotoxicity and cellular uptake of various formulations at pH 7.4 and 5.5 were evaluated on the mammospheres (MS), which were sorted by MCF-7 human breast cancer cell lines and identified to be a CD44^+^/CD24^−^ phenotype. The results of the cytotoxicity assay showed that blank micelles were nontoxic and Thz/PPM exhibited a similar anti-CSC effect on MS compared to Thz solution. Stronger fluorescence signal of Coumarin-6 (C6) was observed in MS treated by C6-loaded PPM (C6/PPM) at pH 5.5. The tumor inhibition rate and tumor weight of the free DOX and Thz/PPM groups were significantly different from those of the other groups, which free DOX and Thz/PPM effectively suppressed breast tumor growth in vivo. The above experimental results showed that Thz/PPM is an ideal and effective pH-responsive drug delivery carrier to a targeted therapy of CSCs.

## 1. Introduction

Breast cancer is the leading cause of cancer deaths among the most common cancers in women worldwide [1,2]. Despite some encouraging progress, many women still experience breast cancer recurrence and metastasis. The only established reason behind this is the potential presence of cancer stem cells (CSCs) [3,4]. CSCs are rare subpopulations of cancer cells associated with tumor initiation, invasion, metastasis, and recurrence, which are highly tumorigenic and resistant to chemotherapy and radiotherapy [5]. In view of this, CSCs have become a new target for anticancer therapy, and CSCs-targeted research with the elimination of CSCs as a therapeutic strategy has an important clinical significance in cancer treatment.

Recent studies show that Thz, which is a piperidine anti-psychotic drug, selectively targets CSC, but is not effective for normal stem cells [6]. This selectivity is due to Thz antagonizing the over expression of dopamine receptors, which are over expressed on the breast CSCs surface [7]. Low concentration of Thz is well tolerated, but larger doses of Thz often lead to adverse reactions, including dry mouth, orthostatic hypotension, vertigo, and nasal congestion [8]. Pigmented retinopathy can occur in large doses of Thz for a long time, and cardiotoxicity may occur in some patients [9]. Therefore, the application of the drug delivery system is needed to reduce the non-specific toxicity of Thz in vivo. Among targeted delivery systems, polymeric micelles are efficient delivery carriers for anti-CSCs drugs [10,11].

Polymer micelles consisting of amphiphilic block copolymers with particle size ranging from 10 to 200 nm are widely used as excellent carriers to enhance drug accumulation within tumors due to the enhanced permeability and retention (EPR) effect [12]. In particular, according to the physiological environment of solid tumors, more stimulus-responsive amphiphilic block polymers have been used as carriers for delivering antineoplastic drugs into tumor tissues [13]. pH-responsive drug release is one of the most important in many stimuli-responsive controlled drug release strategies for cancer therapy [14]. Due to the EPR effect and the low pH value environment in tumor tissues and intracellular lysosomes, different pH-sensitive polymeric micelles can control the release of an anticancer drug in cancer tissues or lysosomes. pH-sensitive polymeric micelles with pKa < 6.8, after being internalized into target cells, aggregated in the endosomes and enter the lysosome (the pH values of early lysosomes and late lysosomes are 6.0 and 6.5, respectively) [15,16]. Therefore, in view of pH-sensitive polymeric micelles, which can be recognized by the tumor or intracellular acid environment and triggered drug release, many pH-sensitive block polymers have been used to construct micelles such as poly(β-amino ester) (PBAE) [17,18].

PBAE is a kind of synthetic polymer material, which can be degraded into non-toxic small molecules by avoiding the accumulation in vivo. Most importantly, PBAE is a pH-sensitive polymer, which is soluble via the protonation of tertiary amino groups in a micro-acid environment. Hence, the acidic environment of the tumor is used as a signal to trigger PBAE to release encapsulated drugs to achieve the goal of tumor-target therapy. In recent years, pH-sensitive polymer micelles composed of PBAE has become a promising drug delivery target carrier to deliver different antitumor drugs including genes [19], small molecule antineoplastic drug [20], and protein and polypeptide drugs [21].

**Scheme 1:**
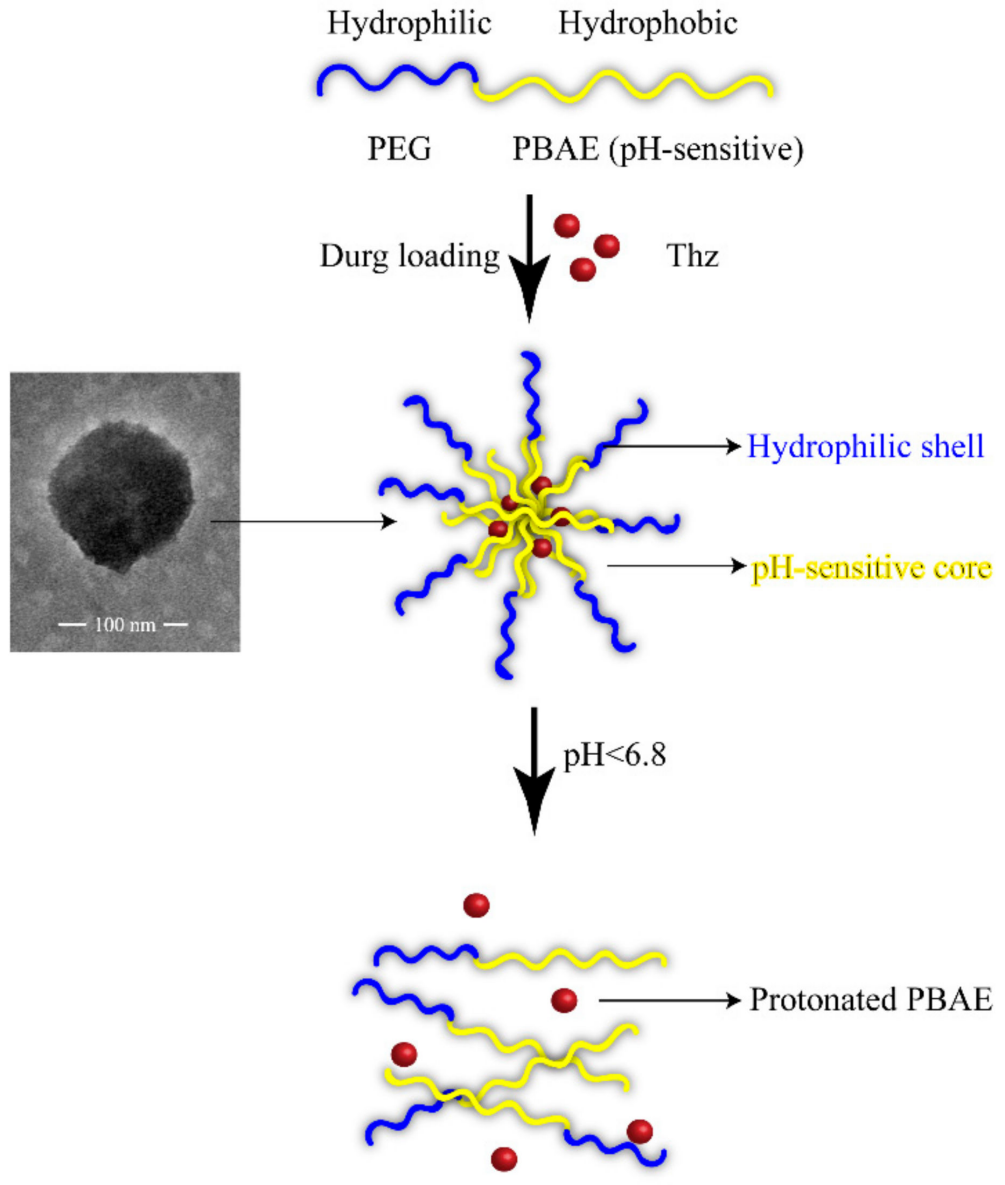
Schematic illustration of drug loading and pH-dependent release of drug from Thz/PPM.

In this study, we aimed to construct a self-assembled micellar delivery system with pH-triggered drug release functions, which provides an effective approach for increasing uptake of CSCs and rapid delivery of the cargo drug into the cytosol, which achieves the goal of targeted therapy of CSCs (Scheme 1) and improves the antitumor efficacy of Dox. Up to now, there are few strategies for the treatment of CSCs by pH-sensitive trigger release. Meanwhile, different from previous reported synthesis strategies of PEG-PBAE, we first synthesized the hydrophobic block to control their pH sensitivity to avoid the cyclization reaction of Michael-type step polymerization. The pH-sensitive PPM was prepared to load Thz that has a cytotoxic effect on breast cancer stem cells (Scheme 1). The Thz/PPM was characterized for particle size, zeta potential, and in vitro release profiles, etc. under different pH conditions. In addition, MS, rich in breast CSCs, were sorted by flow cytometry from MCF-7 cell lines. Meanwhile, the cytotoxicity, MS formation rate, cellular uptake, and endosomal escape of PPM at different pH in vitro were evaluated on MS, respectively. At last, in vivo antitumor efficacies of a different formulation were investigated.

## 2. Materials and Methods

Poly(ethylene glycol) bis (amine) (NH_2_–PEG–NH_2_, Mn 2000) was purchased from Shanghai Ponsure biotechnology Co. Ltd. (Shanghai, China). 1,6-Hexanediol diacrylate (HDD), 1,3-Bis(4-piperidyl) propane (TDP), 4-Methylpiperidine (MP), and thioridazine (Thz) were purchased from Alfa Aesar Chemistry Co. Ltd. (Beijing, China). C6 was obtained from Shanghai Aladdin Biochemical technology Co. Ltd. (Shanghai, China). Pyrene and 3-(4,5-dimethylthiazol-2-yl)-2,5-diphenyl tetrazolium bromide (MTT) were supplied by Sigma-Aldrich Co. (St. Louis, MO, USA). Dulbecco’s modified Eagle medium (DMEM), fetal bovine serum (FBS), and trypsin were purchased from Shanghai ExCell Bio technology Co. Ltd. (Shanghai, China). Human epidermal growth factor (Human EGF) and Fibroblast growth factor (FGF) were obtained from Pepro Tech Inc. (Rocky Hill, CT, USA). All other chemicals were of an analytical grade.

MCF-7 human breast cancer cells were obtained from Shanghai FuHeng biotechnology Co. Ltd. (Shanghai, China). The MCF-7 cells were maintained and supplemented with 10% FBS, in a humidified atmosphere of 5% CO_2_ at 37 °C. To obtain the MS, the adherent MCF-7 cells were digested by trypsin, suspended in DMEM-F12 serum-free medium with a growth factor at a cell density of 3 × 10^4^ cells/mL, and incubated at 37 °C for 20 days. After incubation, the MS were collected with centrifugation at 1000 rpm for 5 min.

Female BALB/c-nude mice (20 ± 2) g were obtained from the Heilongjiang University of Chinese Medicine Laboratory Animal Center (Harbin, China). All animal experiments were carried out under the protocols approved by the ethical committee for biological and medical experimentation of Heilongjiang University of Chinese Medicine (the project identification code: HUCM-LS2018-12-10-101, date of approval: 10 December 2018).

### 2.1. Synthesis of the Block Copolymer

Synthesis of PBAE was synthesized via a Michael step polymerization. TDP (1.1 eq.), HDD (1.0 eq.), and MP (0.1 eq.) were co-dissolved in chloroform. The reactant was stirred at 60 °C for 48 h under nitrogen. After the reaction, chloroform was removed by reduced pressure. Then, the residual reaction residue was precipitated in cold diethyl ether for 12 h, and dried under vacuum for 48 h. Final light-yellow solids were obtained after filtration.

Synthesis of PEG-PBAE was still synthesized via a Michael step polymerization. The mixture of PEG (1 eq.) and PBAE (1 eq.) were stirred at 60 °C for 48 h under nitrogen. At the end of the reaction, the resulting solution was concentrated and transferred to a dialysis bag (Mw 5000) against an excess of distilled water for 48 h. Lastly, the solution was freeze-dried to obtain PEG-PBAE powder.

### 2.2. Characterization of PEG-PBAE

The polymer structure of PBAE and PEG-PBAE were characterized by ^1^H NMR spectroscopy (400 Hz, ARX-300, Bruker, Fllanden, Switzerland) with deuterated chloroform (CDCl_3_) as solvent and tetramethylsilane (TMS) as an internal standard.

The molecular weight and molecular weight distribution of the copolymer were determined by gel permeation chromatography (GPC) with tetrahydrofuran (THF) as a mobile phase and polystyrene as the standard sample.

The buffering capacity of PEG-PBAE copolymers was determined by the acid-base titration method. PEG-PBAE (30 mg) was dissolved in 30 mL of deionized water. The pH of copolymer solution was first adjusted to 2 with 0.1 M HCl, and then titrated to 11 with 0.01 M NaOH given in various volume increments. Acid–base titration profiles of the copolymer were made with the consumed volume of NaOH and the corresponding pH.

The CMC of the PEG-PBAE copolymer was determined with a fluorescent spectroscopy (LS55, Waltham, MA, USA) with pyrene as a hydrophobic probe [22]. Additionally, 1 mL of pyrene in acetone (6.0 × 10^-5^ M) was added to each glass bottle, and then acetone in each glass vial was vaporized at 60 °C. The solutions of blank PEG-PBAE with a gradient concentration ranging from 0.5 to 100 μg/mL were added to each vial, and all the samples were stored in the dark for 24 h. The peak intensities at 335 and 336 nm from the excitation spectra were analyzed with an emission wavelength at 397 nm for calculating the CMC.

### 2.3. Preparation of Thz/PPM

The thin-film dispersion method was used to prepare the drug-loaded micelles [23]. Firstly, PEG-PBAE and Thz with a feed weight ratio of 10:3 were dissolved in 10 mL of acetone. Then, a homogeneous thin-film was obtained by removing acetone in a vacuum dryer for 12 h. Secondly, 10 mL of deionized water was added to disperse the obtained thin film layer, which was completely hydrated by ultrasonication. Eventually, after centrifugation at 10,000 rpm for 15 min, the supernatant was continued to be filtered with a 0.45-μm Millipore filter to eliminate the insoluble drug. Then the final solution of PPM was obtained. Blank polymeric micelles and C6/PPM were prepared by the same method as mentioned above.

### 2.4. Characterization of Thz/PPM

A transmission electron microscopy (TEM) (JEM-2100, Jeol, Tokyo, Japan) was used to estimate the morphology of polymeric micelles. The sample solution was added to the special copper mesh for transmission electron microscopy, and stained by phosphotungstic acid for 5 min. When the copper mesh dried at room temperature, the sample was observed and taken photos by using TEM.

A particle analyzer (Zetasizer nano-ZS90, Malvern, UK) was used to assess the particle sizes and Zeta potential of the polymeric micelles, which were responsive to pH. The pH values of the sample solution of blank micelle were adjusted with HCl (0.1 M), and the sample was incubated for 15 min.

The drug loading content (LC) and the drug encapsulation efficiency (EE) were calculated with the ratio of the content of drugs in Thz/PPM to the total weight of Thz/PPM and supporting quality of drug, respectively. A UV-spectrophotometer (UV-2600, Shimadzu, Kyoto, Japan) was used to measure the Thz concentration at 301 nm.

### 2.5. pH-Dependent Drug Release from Thz/PPM

A dialysis bag diffusion method was used to evaluate the in vitro release of Thz from Thz/PPM [24]. A dialysis bag (Mw 3500) containing 2 mL of Thz/PPM solution was placed in 35 mL fresh PBS (pH 7.4, 6.8, or 5.5 containing 0.5% sodium dodecyl sulfonate (SDS)), and shaken at a speed of 100 rpm at 37 °C. At pre-set time intervals, 1.0 mL of the release solution was pipetted and replaced with the same volume of fresh buffer. The concentration of Thz releasing into PBS was analyzed with the HPLC method mentioned previously. The cumulative release (*Cr*) percentage of Thz from the PPM was calculated using the following Equation (1),

(1) $$\mathrm{Cr}=\frac{V_{e}\sum_{i-1}^{n-1}C_{i}+V_{0}C_{n}}{m_{\mathrm{drug}}}\times 100\%$$

where *Cr* was a cumulative release of Thz, *V_e_* was the replacement volume of PBS, *C_i_* was drug concentration released during the *i*th replacement sampling, *V_0_* was the volume of the release mediums, *C_n_* was drug concentration in the release medium at the *n*th sample, and *m_drug_* was the content of Thz in drug-loaded micelles.

### 2.6. In Vitro Cytotoxicity Assays

The in vitro cytotoxicity of blank PPM and Thz/PPM was evaluated by the MTT assay. MS suspension with a density of 1 × 10^4^ cells per well were seeded on 96-well plates in 90-µL culture medium, and incubated for 24 h. Then, free Thz, blank micelles, and Thz/PPM, with a gradient concentration of Thz, were used to treat the MS in PBS with a pH 7.4 or 5.5, and incubated for 48 h. After incubation, a 20-µL MTT solution (5 mg/mL) was added into each well to a further incubation for 4 h. The culture medium was removed and 100-µL triplex solution (10% SDS, 5% isobutanol and 0.01 M HCl) was added to dissolve the formazan crystals. The absorbance at 570 nm was recorded by using a microplate reader (Synergy H1, Winooski, VT, USA). The following Equation (2) was used to calculate cell viability (%),

(2) $$\mathrm{Cell}\;\mathrm{viability}=\frac{A_{\mathrm{sample}}}{A_{\mathrm{control}}}\times 100\%$$

where *A_sample_* and *A_control_* were the absorbance of cells in the absence and in the presence of sample treatment, respectively. The IC_50_ values of different groups were calculated using origin statistics software program (Origin 9.1, OriginLab, Northampton, MA, USA). 

### 2.7. Experiment of MS Formation Rate In Vitro 

The anti-breast cancer stem cell activity in vitro was evaluated by the rate of MS formation of ex vivo tumor cells. The removed tumor tissue was sterilized, shredded, and digested to obtain a single cell suspension. After the cells were attached, the stem cells are cultured according to the cell culture method mentioned in the text. After seven days of culturing, the number of MS and the morphology of the MS were recorded.

### 2.8. In Vitro Cellular Uptake 

The cellular uptake of C6 was observed by a fluorescence microscopy (Lelca Microsystems™ DM IL LED, Wetzlar, Germany). MS with a density of 2.5 × 10^4^ cells per well were seeded on 6-well plates, and incubated for 12 h at 37 °C. Then the culture medium was removed. The MS were cured with C6/PPM for 0.5, 2, and 4 h in serum-free medium with pH 7.4 and 5.5. At the end of incubation, the cells were centrifuged at 1000 rpm for 5 min and the supernatant was quickly poured out. The cells were washed with cold PBS to terminate the cellular uptake. The cells were observed and taken photos by the fluorescence microscopy. A flow cytometer (Guava EasyCyte™ 8HT, Darmstadt, Germany) was used to quantitatively analyze the cellular uptake amount.

### 2.9. Study of Cellular Uptake Mechanism

MS was digested to prepare a single cell suspension and seeded in 6-well plates at a cell density of 1 × 10^5^ cells. After incubation for 12 h at 37 °C, uptake inhibitors including 10 μg/mL chlorpromazine (CPM), 40 μg/mL colchicines (CC), and 5 μg/mL filipin (FLP) were added. After incubation for 30 min, with the inhibitor-containing medium discarded, Thz/PPM (equivalent to containing 5 μg/mL Thz) was added and continued to be incubated for 2 h at 37 °C, 5% CO_2_. The cells without the uptake inhibitor were part of the control group. After collecting the cells, the cells were assayed by flow cytometry.

### 2.10. Endosomal Escape of Thz/PPM 

The MS suspension, which was obtained by trypsinization, were seeded on a 6-well culture plate containing a cover glass at 1 × 10^5^ cells/well in 2 mL of culture medium, and incubated for 24 h. Additionally, 2 mL of C6/PPM solution was added and incubated for 10 min, 30 min, 1 h, and 2 h at 37 °C. After the incubation, the cells were washed 3 times with PBS and stained with 100 nM Lyso-Tracker Red lysosomal dye for 10 min. After washing with PBS, 200 μL of Hoechst 33258 solution were add for 10 min. Lastly, the cells were washed 3 times with PBS, fixed in 4% paraformaldehyde for 30 min, and observed by CLSM. In addition, in order to investigate the release of polymer micelles in the intracellular lysosomal acidic environment. MS cells were pre-incubated with chloroquine for 1 h and then C6/PPM was added for 2 h. After the incubation, the cells were observed by CLSM.

### 2.11. Xenograft Tumor Model

The breast tumor xenograft model was established using a logarithmic growth phase of MCF-7 cells. MCF-7 cells were trypsinized and resuspended in 0.9% saline. The cell concentration was approximately 5 × 10^6^ cells/200 μL, and 0.2 mL of the cell suspension was inoculated into the left breast pad of female Balb/c nude mice. Tumor growth was observed every three days. Treatment started after the tumor volume reached 50 to 100 mm^3^.

### 2.12. In Vivo Antitumor Efficacy

The MCF-7 xenograft tumor-bearing nude mice were established as described above. The mice were randomly divided into five groups (six mice per group) when the tumor grew to about 100 mm^3^. The five groups were intravenously injected with saline, blank PPM, Thz solution, Dox and Thz solution, and Dox solution and Thz/PPM, respectively (Dox: 5.0 mg/kg). After 22-day tests of different formulations, these mice were sacrificed, and then tumor tissues were harvested and weighted. The tumor inhibition rates (TIR %) were calculated by the following Equation (3),

(3) $$\mathrm{TIR}\;\%=\frac{\mathrm{Wc}-\mathrm{Wt}}{\mathrm{Wc}}\times 100\%$$

where Wc and Wt represent the tumor weight of the control group and the tested groups, respectively.

### 2.13. Statistical Analysis

All the experiments were repeated at least three times and the data were expressed as mean ± standard deviation (SD). The difference among the groups was evaluated by analysis of variance (ANOVA) among ≥3 groups or a Student’s *t*-test between 2 groups. A *p*-value less than 0.05 was considered statistically significant.

## 3. Results and Discussion

### 3.1. Synthesis of PEG-PBAE Copolymers

**Scheme 2:**
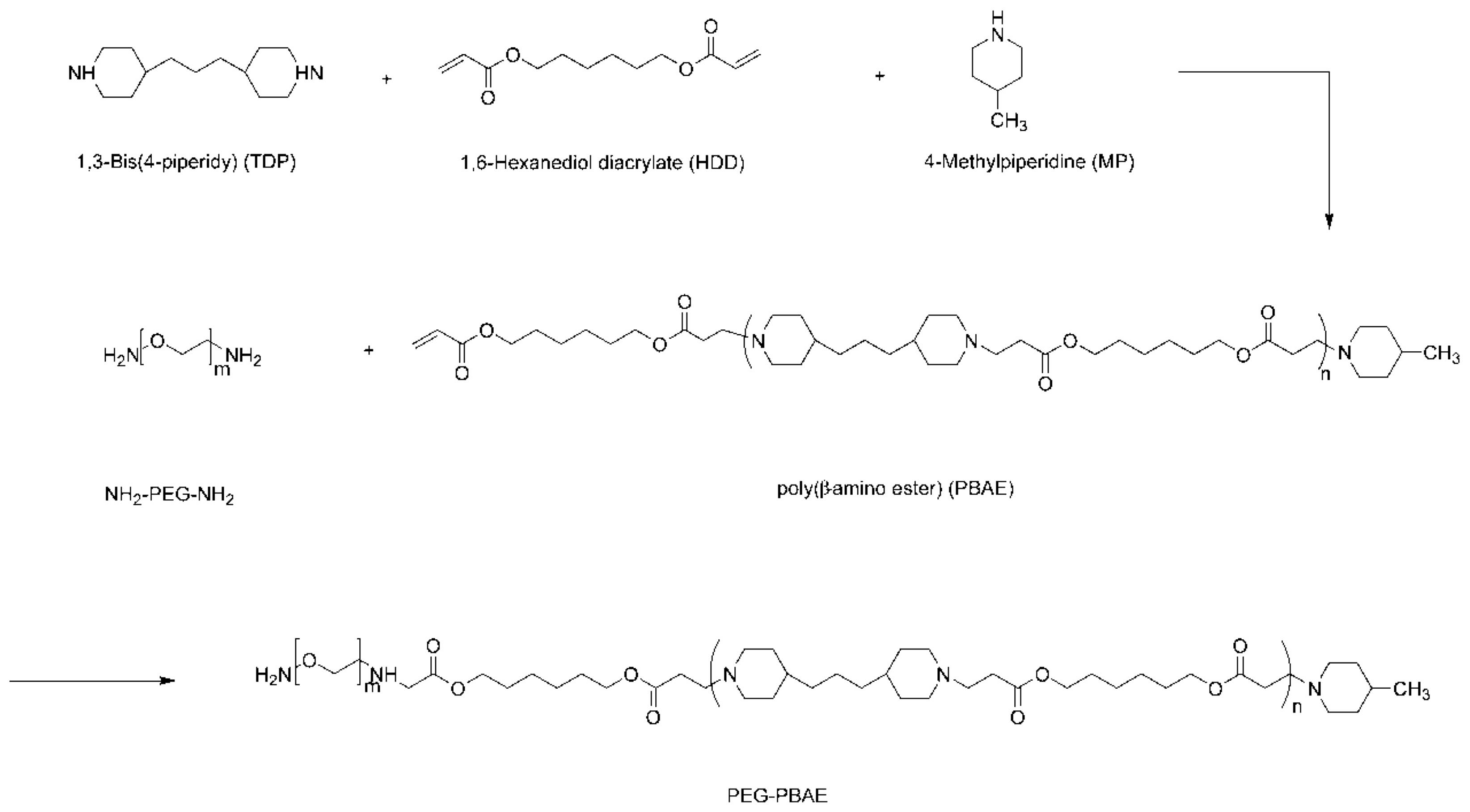
Synthetic route of PEG-PBAE.

**Figure 1:**
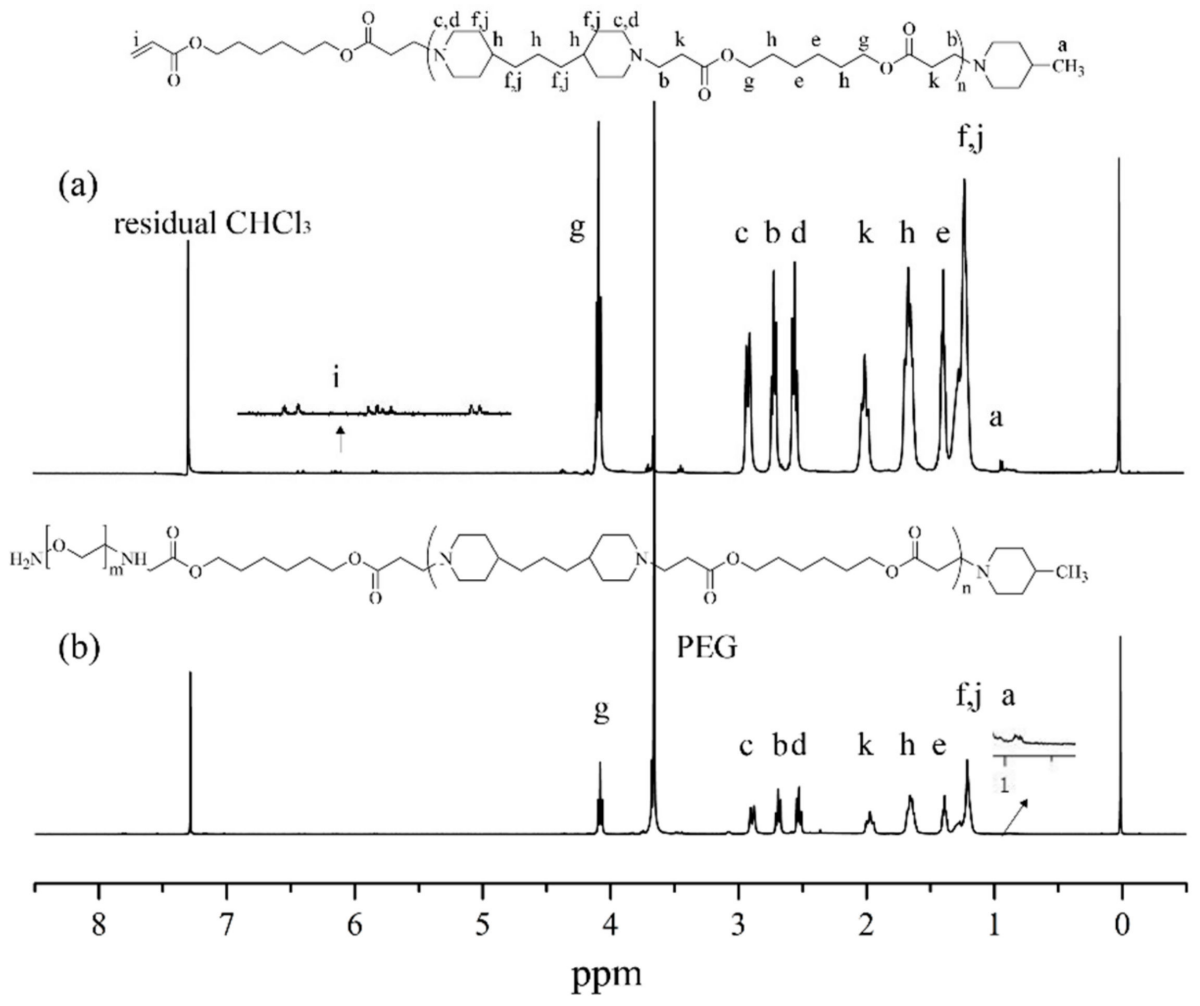
^1^H NMR spectra of PBAE (**a**) and PEG-PBAE (**b**).

The synthetic route for PEG-PBAE was entirely illustrated in Scheme 2. The ^1^H NMR was used to evaluate the synthesis of PEG-PBAE. The ^1^H NMR spectrum of PBAE and PEG-PAE were shown in Figure 1. As expected in Figure 1a, the peaks at 0.94, 2.69, and 2.89 ppm were the characteristic peaks of PBAE, and the typical peaks of the hydrogen of the acrylate unit were at 5.79, 6.12, and 6.38 ppm, which proved that PBAE was successfully synthesized. In Figure 1b, the characteristic peak of PEG was observed at 3.72 ppm, and the typical peaks of the acrylate segment were not observed in the ^1^H NMR spectrum of PEG-PBAE. It indicated that the terminal acrylate unit of PBAE was successfully reacted with the amino group on the PEG chain. These results of ^1^H NMR spectrum of PBAE and PEG-PAE were in line with the reported literature [25,26]. In this synthetic, the introduction of MP to the terminal group of the PBAE was for preventing the cyclization reaction of Michael-type step polymerization. In addition, the PBAE and PEG-PBAE structures were further verified by Infrared Spectroscopy (see Figure S1).

### 3.2. Characterization of PEG-PBAE Copolymers

GPC results of PBAE and PEG-PBAE copolymers were shown in Table 1. Meanwhile, as shown in Table 1, the pKa of PBAE and PEG-PBAE polymers were 6.6 and 6.7, respectively. Acid–base titration profiles of the PEG-PBAE copolymer with NaCl as a control was shown in Figure S2.

**Figure 2:**
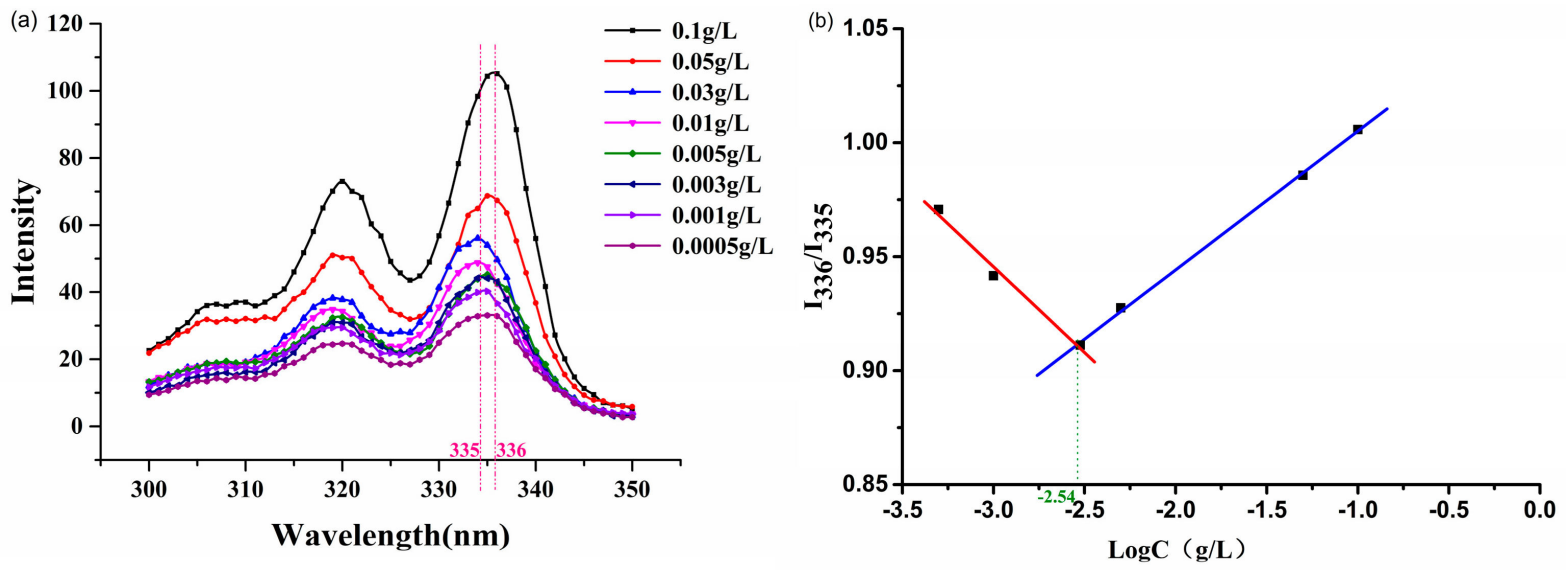
(**a**) Emission spectra of pyrene as a function of copolymer concentration and (**b**) fluorescence intensity ratio of I_336_/I_335_ ratio from emission spectra vs. log concentration of the copolymers at pH 7.4.

The CMC of the PEG-PBAE copolymers was measured by the change of fluorescence spectrum of pyrene [27]. In Figure 2a, the fluorescence intensity increased with the increments of PEG-PBAE concentration, and, in the excitation spectra of pyrene, the peak shifted from 335 to 336 nm. The fluorescence intensity ratio (I_336_/I_335_) of the pyrene is a function of copolymer concentration, which was illustrated in Figure 2b. The CMC value of PEG-PBAE copolymers was 2.49 μg/mL and was extraordinarily lower than that of other low molecular weight surfactants [28]. The low CMC of the copolymers indicated that the PEG-PBAE copolymers had self-assembly ability in vitro, and the PPM formed by PEG-PBAE copolymers had very good dilution stability in blood circulation [29].

### 3.3. Characterization of Thz/PPM

**Figure 3:**
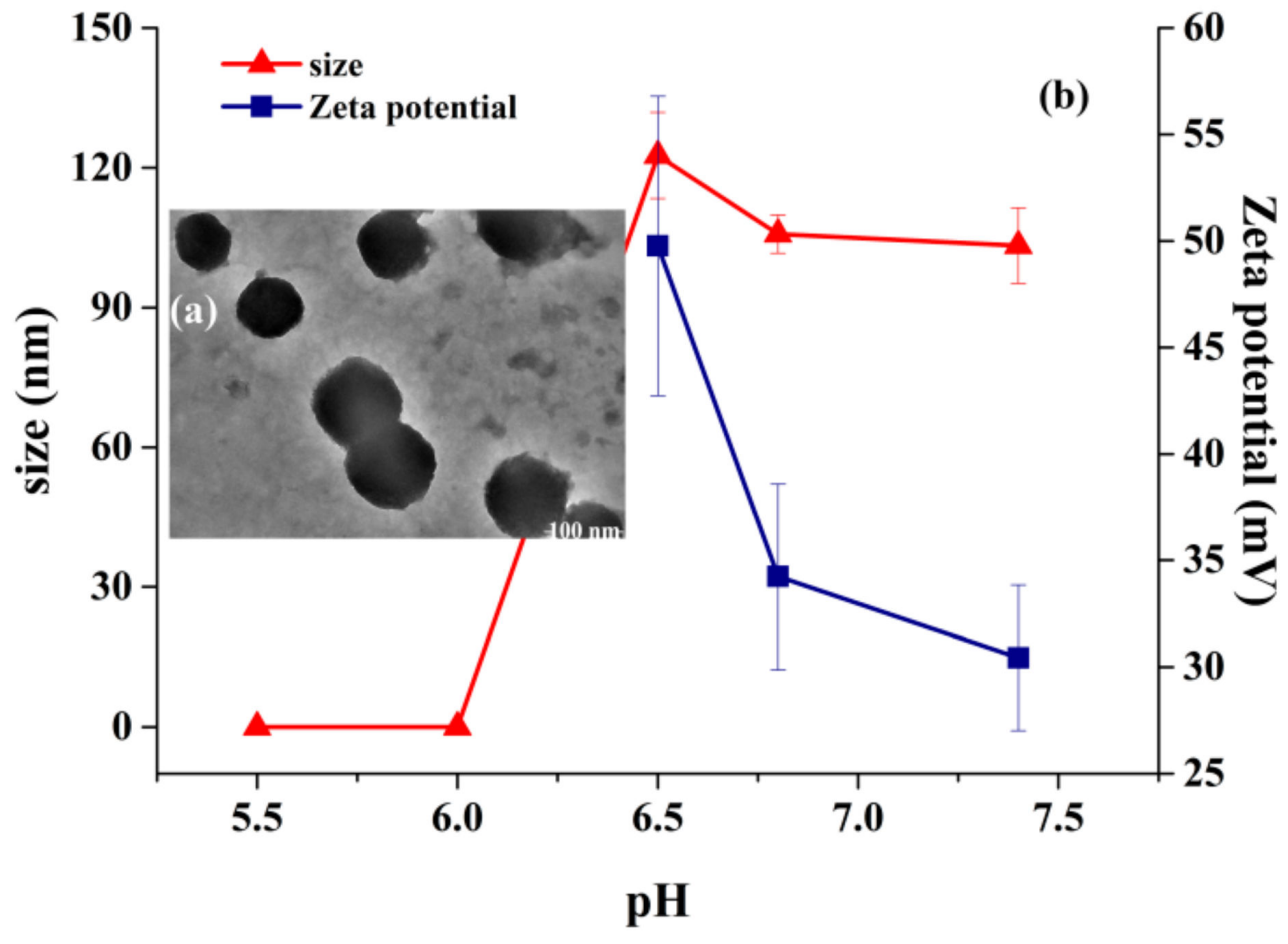
(**a**) TEM images of PEG-PBAE (scale bar, 100 nm) and (**b**) size distribution and Zeta potential changes of blank PEG-PBAE at different pH values of 7.4, 6.8, 6.5, 6.0, and 5.5 (*n* = 3).

The TEM image showed that the Thz/PPM was monodisperse spheres without clear adhesion (Figure 3a). For further studying the pH-sensitivity of the micelles, Figure 3b presented the particle size of the blank micelle with pH sensitivity at different pH values. In Figure 3b, when the media pH value increased over 6.8, the particle size of block copolymer micelles was 105.77 ± 4.10 nm and did not change significantly in solution. It was proven that micelles had good structural stability at blood circulation pH (7.4). Furthermore, when the pH value dropped from 6.8 to 6.5, the micelle size increased slightly, which indicated that, due to the partial protonation of PBAE block, the charge repulsion in the core of the micelle promoted the expansion of the micelle, which resulted in the increase of particle size. However, when the pH value continued to decrease below 6.0, the micelles size could no longer be detected as the tertiary amine part of PBAE that was completely protonated. This suggests that the micelle structure no longer exists. The above results demonstrated that the Thz/PPM exhibited a clear pH-responsive feature. Moreover, small and homogeneous particles (<200 nm) of the Thz/PPM were able to reduce phagocytosis of the reticuloendothelial system, which supplies excellent passive tumor-targeting through the EPR effects [30].

As revealed in Figure 3b, the Zeta potential of the Thz/PPM at pH 7.4 was 30.4 ± 3.41 mV, and, with the decrease of pH, the Zeta potential increased to 49.7 ± 7.02 mV at pH 6.5, which was due to protonation of the tertiary amine units in PBAE. However, when the pH dropped below 6.5, due to the complete protonation of the PBAE segment, which resulted in the absence of insoluble particles in the solution, the Zeta potential could not be measured.

According to the calculation method mentioned above, when the ratio of the drug to polymer was 3:10, the LC was 15.5%, and the EE was more than 85%.

### 3.4. pH-Dependent Release of the Drug

**Figure 4:**
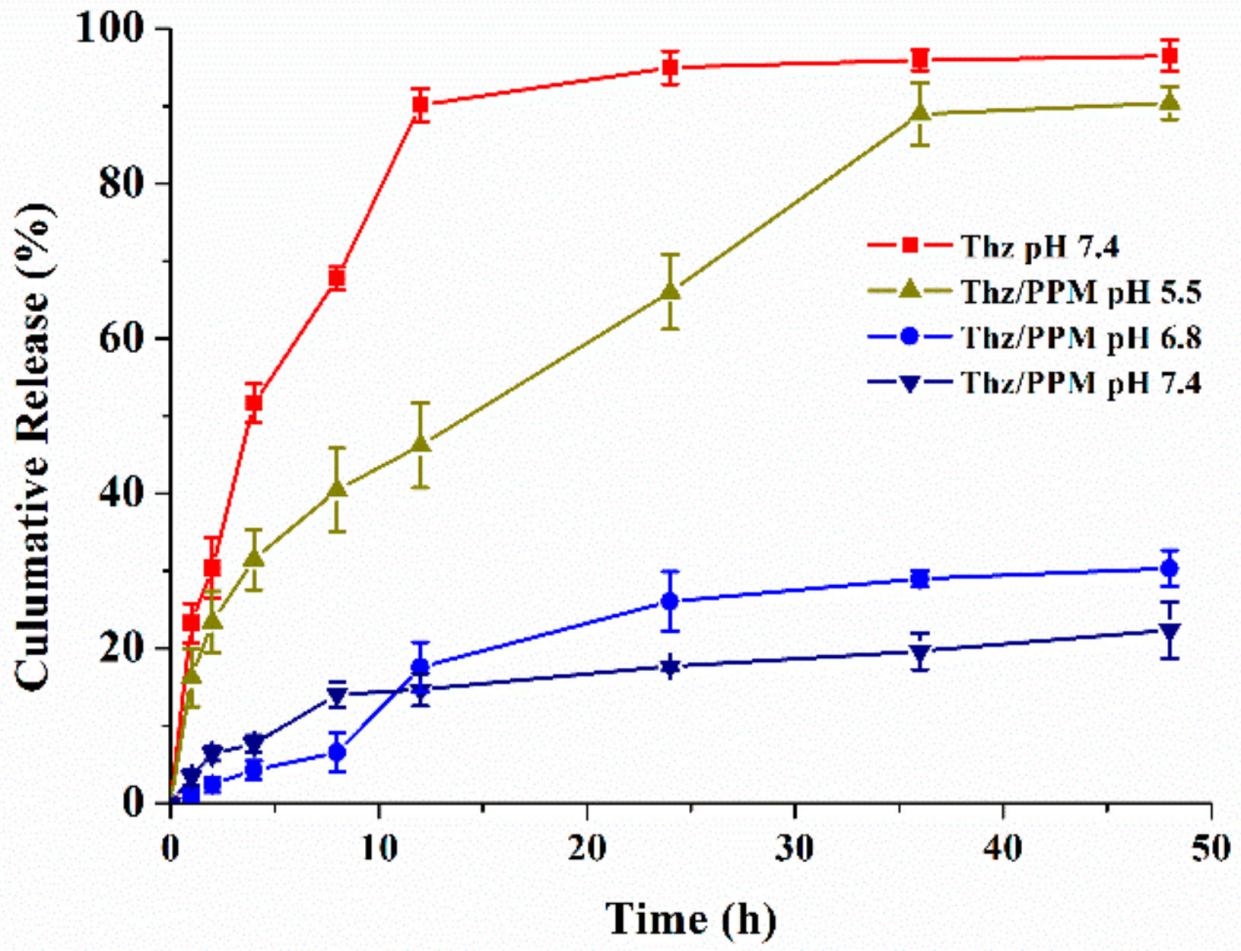
Release profile of Thz from Thz/PPM under different pH values (7.4, 6.8, and 5.0) in vitro (*n* = 3).

The studies of the in vitro release behaviors of the Thz/PPM in release medium of different pH values were shown in Figure 4. First, free Thz could be completely released in about 12 h, and the release rate of Thz could be significantly delayed by encapsulating Thz in PPM (*p* < 0.01). This was because Thz was encapsulated in the hydrophobic nucleus of PPM. With the dissolution and degradation of PPM, the drug could be released slowly. Second, in the release medium of pH 7.4 (physiological conditions), there was no clear burst of Thz in PPM, which indicates that the PPM encapsulated most of the hydrophobic Thz into micelles’ core due to the hydrophobic interaction. Under this pH condition, Thz/PPM exhibited a relatively stable release characteristic of releasing about 25% of Thz at 24 h and only about 30% at 36 h, which suggested that the micelles were well-structured under physiological conditions. Lastly, in the pH 5.5 (acidic tumor environment) release medium, the release behavior of Thz in Thz/PPM was affected by the pH value. The accelerated release rate of Thz/PPM was about 31% in 4 h, 66% in 24 h, and 88% in 36 h, which was significantly higher than that in the pH 7.4 release medium at the same time (*p* < 0.01). This indicated that the micelle structure did not exist, and the drugs were nearly fully released in pH 5.5 release medium solution. Lastly, at pH 6.8, the release of Thz from Thz/PPM was incomplete, which indicates that the micellar structure was not destroyed in the acidic environment of the tumor tissue and Thz was delivered into the cells. Drug release of Thz/PPM has significant pH sensitivity. It is speculated that the reason may be that the polymer micelle structure is stable at pH 7.4, which could delay drug release. In the pH 5.5 environment, the protonation of the amino group in the carrier PBAE caused the PBAE to dissolve. Electrostatic repulsion between the PBAE blocks of the micelle core would cause the micelle structure to be destroyed and the drug to be released quickly. The above results indicated that hydrophobic and pH-sensitive PBAE segment of PPM could control the release of Thz release based on the differences in the pH environment. This is particularly important for the release of Thz from polymeric micelles in cellular pH environments of CSCs.

### 3.5. In Vitro Cytotoxicity Assays

**Figure 5:**
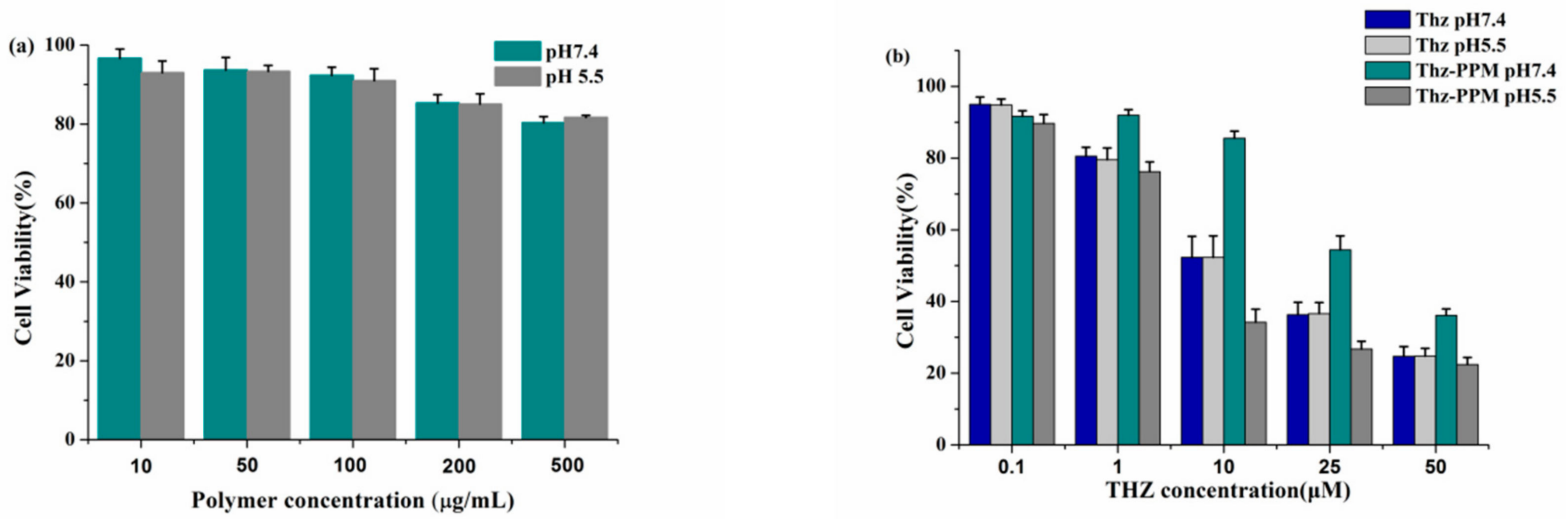
(**a**) Relative cell viabilities of MS cells incubated with blank micelles, (**b**) free Thz and Thz/PPM at pH 7.4 and 5.5 for 48 h (*n* = 3).

MS cells were isolated by serum-free method, and MS cells exhibited the phenotypic characteristics of CD44^+^/CD24 (see Figure S3 and Figure S4). At pH 7.4 and pH 5.5, the cell viability of the blank PPM and Thz/PPM against MS was studied after incubation for 48 h. As expected, the blank PPM consisting of low toxic PEG and PBAE blocks segments showed little toxicity in MS cells at pH 7.4 and 5.5 for 48 h. The cell viability of blank PPM was higher up to 80% with a polymer concentration of 500 µg/mL in MS (Figure 5a). For drug-loaded micelles, the free Thz solution was set as the control, as shown in Figure 5b, the Thz solution and Thz/PPM showed dose-dependent cytotoxicity in MS cells. With the increase of Thz concentration in the preparation, the cytotoxicity of each preparation increased. The cytotoxicity of Thz/PPM at pH 5.5 (IC_50_ = 21.43 ± 3.74 μM/mL) was significantly higher than that (IC_50_ = 47.91 ± 5.11 μM/mL) at pH 7.4 (*p* < 0.05). There was no significantly different cytotoxicity between Thz solution at pH 5.5 (IC_50_ = 22.683 ± 1.96 μM/mL) and Thz solution at pH7.4 (IC_50_ = 23.50 ± 4.66 μM/mL) (*p* > 0.05). Compared with the cytotoxicity of Thz solution, the cytotoxicity of Thz/PPM at pH 7.4 was significantly lower than Thz solution (*p* < 0.01), but there was no significantly different cytotoxicity between Thz/PPM at pH 5.5 and Thz solution (*p* > 0.05). In the light of the results of in vitro pH-dependent release of Thz studies, at the same time, Thz released from Thz/PPM at pH 5.5 was clearly more rapid than that at pH 7.4. Furthermore, the released amount of Thz in 48 h was equivalent to that of Thz/PPM at pH 5.5. Therefore, Thz/PPM at pH 5.5 showed higher cytotoxicity than that at pH 7.4, but was similar to cytotoxicity with Thz solution.

### 3.6. Experiment of MS Formation Rate In Vitro

**Figure 6:**
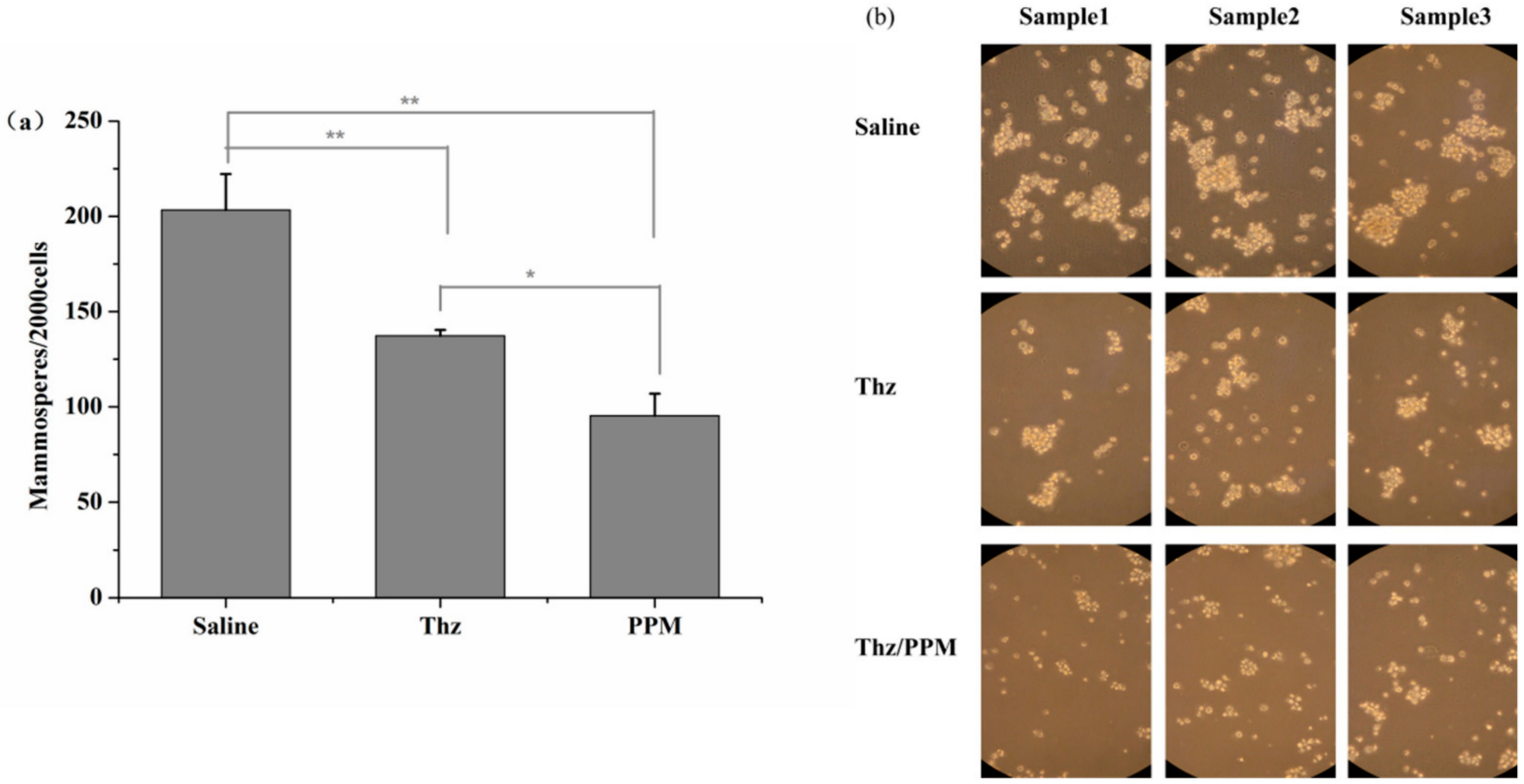
In vitro anti-CSCs activity. (**a**) Mammospheres number and (**b**) images of optical micrograph (magnify 200 times). * *p* < 0.05, ** *p* < 0.01.

Figure 6 showed results of the MS formation rate and morphology isolated tumor cells after treatment with different drugs. As shown in Figure 6b, for the saline group, breast tumor tissue cells can be cultured in suspension to form large and dense MS. In Figure 6a, compared with the saline group, the number and the volume of MS formed in the free Thz decreased, which indicates that free Thz had a good inhibitory effect on MS cells, and the formation rate was about 67% (*p* < 0.01). The number and volume of MS in the Thz/PPM were also significantly reduced, which indicated that the Thz/PPM had significant inhibition on MS cells compared with the saline group (*p* < 0.01) and the free Thz (*p* < 0.05).

### 3.7. In Vitro Cellular Uptake

**Figure 7:**
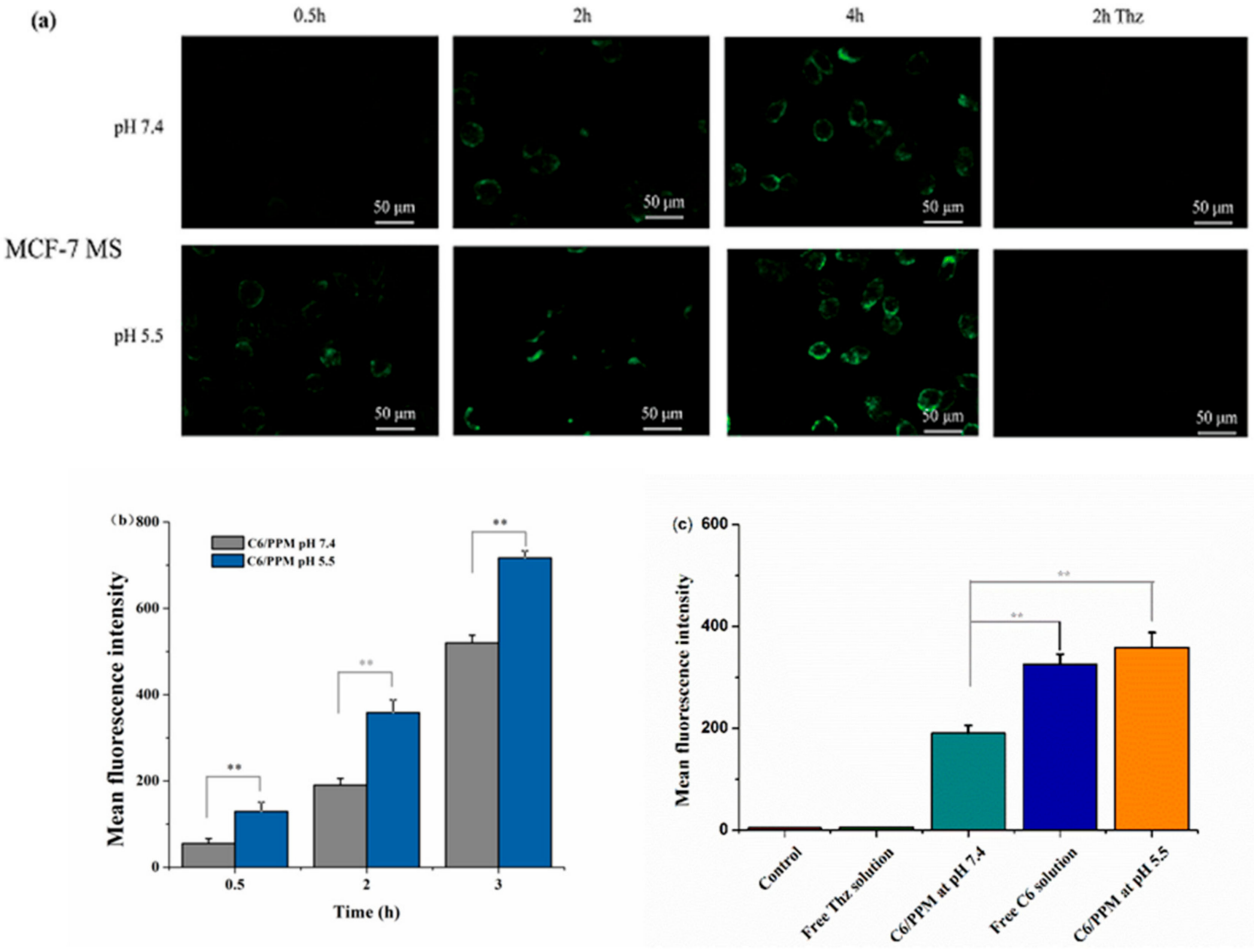
(**a**) Confocal images of MS incubated with C6/PPM at pH 7.4 and 5.5 for 0.5, 2, and 4 h. (**b**) Fluorescence intensity of MS incubated with C6/PPM at pH 7.4 and 5.5 for 0.5, 2, and 4 h as determined by flow cytometry (*n* = 3). (**c**) Fluorescence intensity of MS incubated with C6 solution and C6/PPM at pH 7.4 and 5.5 for 2 h as determined by flow cytometry (*n* = 3). Scale bar, 0.05 mm. ** *p* < 0.01.

Fluorescence microscopy results of drug-induced PPM to enhance cellular uptake at different times and pH values were shown in Figure 7a. The intracellular fluorescence intensity of C6 was very weak after 0.5 h incubation, increased slightly after incubation for 2 h, and increased markedly after incubation for 4 h. This indicated that the behavior of C6 in cellular increased uptake was time-dependent. More importantly, at the same time, the fluorescence of MS remedied with C6-PPM at pH 5.5 was more intense than that at pH 7.4, which indicated that the PPM was capable of effectively delivering drugs into the cytoplasm to improve intracellular uptake due to pH-responsive release.

Figure 7b represented the results of cellular uptake analyzed by using flow cytometry. As mentioned above, the behavior of cellular uptake was time-dependent. The mean fluorescence intensity of C6/PPM at different pH values showed a great increase at 0.5, 2, and 4 h. Moreover, compared with pH 5.5, the uptake amount of C6/PPM was evidently less under pH 7.4, which also demonstrated that cellular uptake behavior of C6 in PPM was pH-dependent. Compared with C6 solutions, the C6/PPM at pH 7.4 revealed less cellular uptake (*p* < 0.01) (Figure 7c). It may be due to the sustained drug release characteristics of pH-sensitive micelles at different pH values, and the different cell uptake pathways of C6/PPM and C6 solution [31]. However, compared with the amount at pH 7.4, the cellular uptake amount of C6/PPM was significantly higher at pH 5.5 (*p* < 0.01), which was consistent with the results of cytotoxicity studies of Thz/PPM mentioned above.

### 3.8. Study of Cellular Uptake Mechanism

**Figure 8:**
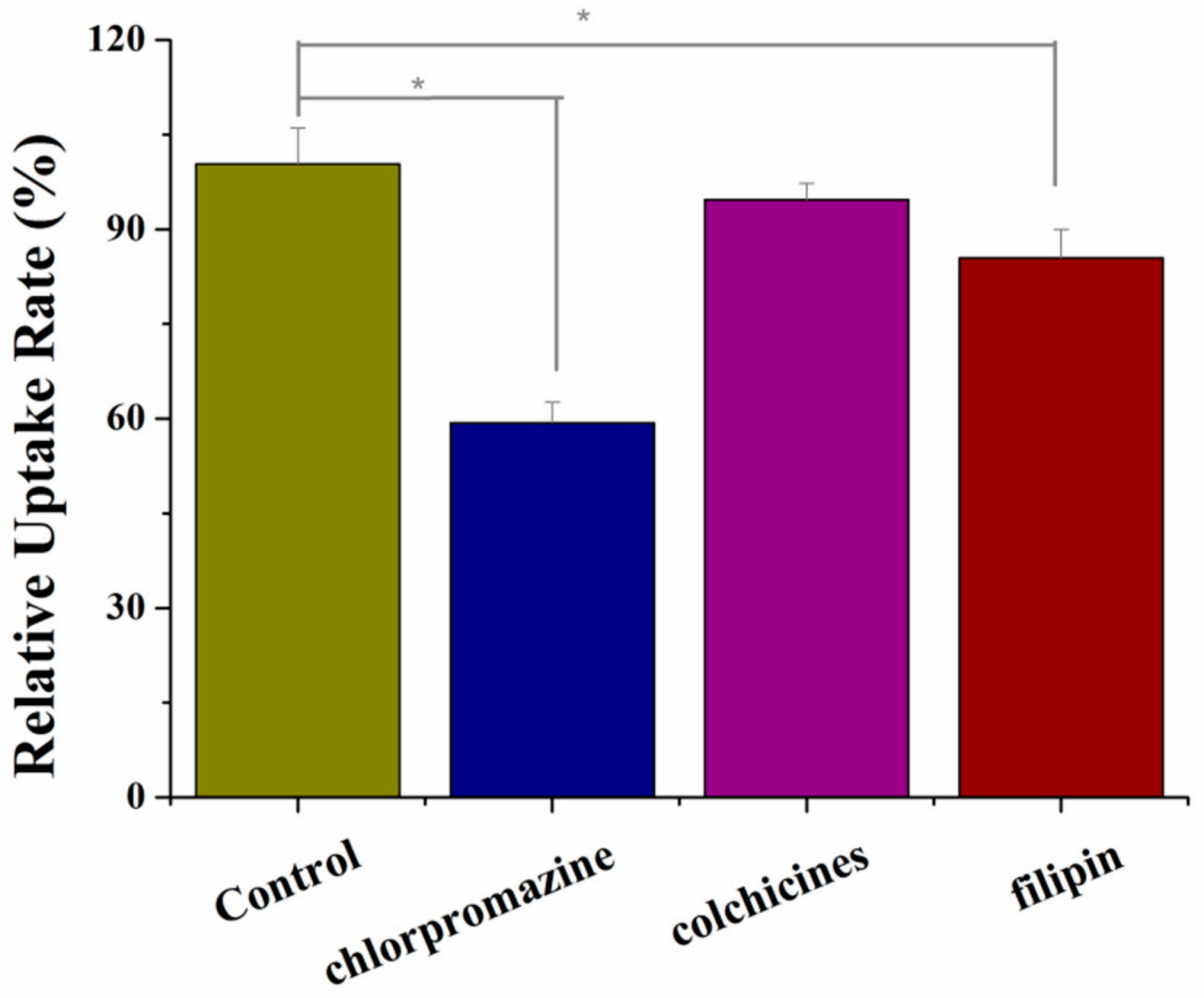
Effects of inhibitors on the internalization of Thz/PPM in MS (*n* = 3). * *p* < 0.05.

The mechanism of uptake was mainly to investigate the effects of different endocytosis inhibitors on cellular uptake. In this experiment, chlorpromazine (CPM) cleaved clathrin and AP-2 protein complexes from the cell surface, allowing them to assemble into endosomes to specifically inhibit clathrin-mediated endocytosis [32]. Filipin (FLP) is a caveolae-mediated endocytosis inhibitor [33]. Colchicines (CC) are selective inhibitors of the macropinocytosis pathway [34]. The results of cellular uptake of these three different inhibitors were shown in Figure 8. CC had little effect on cellular uptake, which indicates that endocytosis of Thz/PPM was not through the macropinocytosis. When MS was treated by CPM and FLP, the cellular uptake amount decreased to 59.3% and 85.4% of the control group, respectively (*p* < 0.05). It was indicated that the uptake mechanism of Thz/PPM polymer micelles in MS cells was mainly that clathrin and caveolin-mediated endocytosis were involved in the uptake of PPM micelles by cells, and clathrin plays a major role.

### 3.9. Endosomal Escape of Thz/PPM

**Figure 9:**
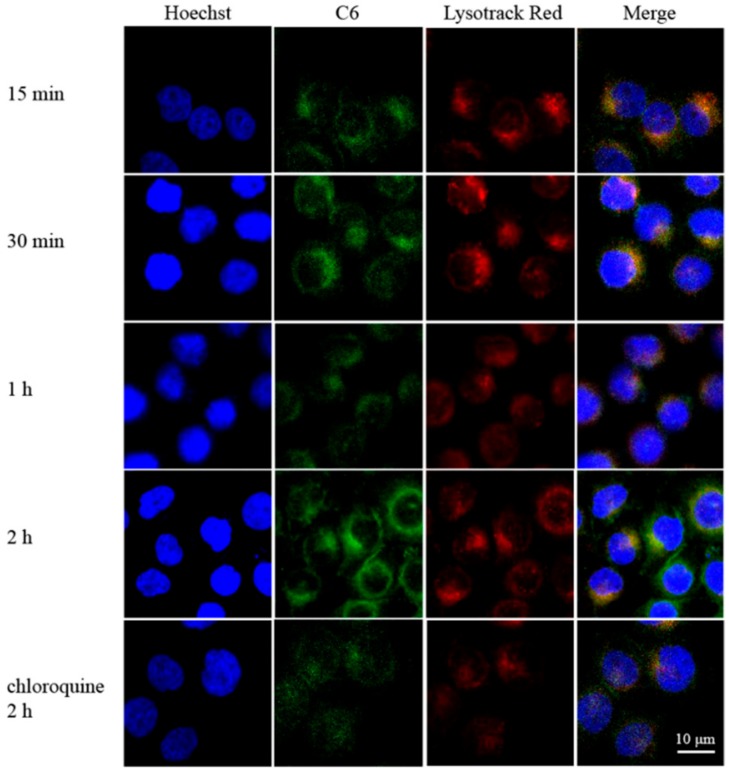
The CLSM images of the MS incubated with C6/PPM for 15 min, 30 min, 1 h, 2 h, and with chloroquine for 2 h. Blue, green, and red colors indicate Hoechst 33258, C6, and LysoTracker Red, respectively. Scale bars, 0.01 mm.

It was critical to escape from lysosomes for drugs that need to reach the cell matrix or nucleus to work [35]. Drug delivery of C6/PPM in MS cells was observed by CLSM. As shown in Figure 9, after incubation for 10 min and 30 min, the cytoplasm showed a distinct yellow fluorescence (yellow fluorescence was formed by the overlap of C6 and lysosomes). This phenomenon indicated that C6 entered the cells and accumulated mainly in lysosomes. After 1 h of incubation, the yellow fluorescence gradually decreased, which indicated that part of the C6 escaped from the lysosome and released into the cytoplasm. When incubated for 2 h, it was clear that C6 escaped from the lysosome, mainly distributed in the cytoplasm. This result indicated that the micelles entered the cells through the endocytosis and then transported through the lysosome. Then, in the acidic environment of the lysosomes, the PBAE could be dissolved by the protonated protonation, which destroyed the micelle structure. In addition, the protonation of amino groups made PBAE soluble. The soluble and cationic PBAE promoted the positive charges to interact with the negatively charged endosome membrane and facilitate escape [36]. Therefore, the occurrence of protonation also caused the lysosomal membrane to rupture and the drug to be released into the cytoplasm. At the same time, when chloroquine was added to treat the cells, the acidic environment of lysosomes was inhibited. As shown in Figure 9, the drug was mainly localized in the lysosome, and almost red fluorescence was not observed in the nuclear region. This phenomenon again demonstrated that the micelles were pH-sensitive enough to release the drug in a lysosomal acidic environment. It is important to note that, by responding to the acidic environment of lysosomes, the potential applications of pH-sensitive delivery not only in cancer therapy but also in certain other fields is significant [35].

### 3.10. In Vivo Anti-Tumor Efficacy

**Figure 10:**
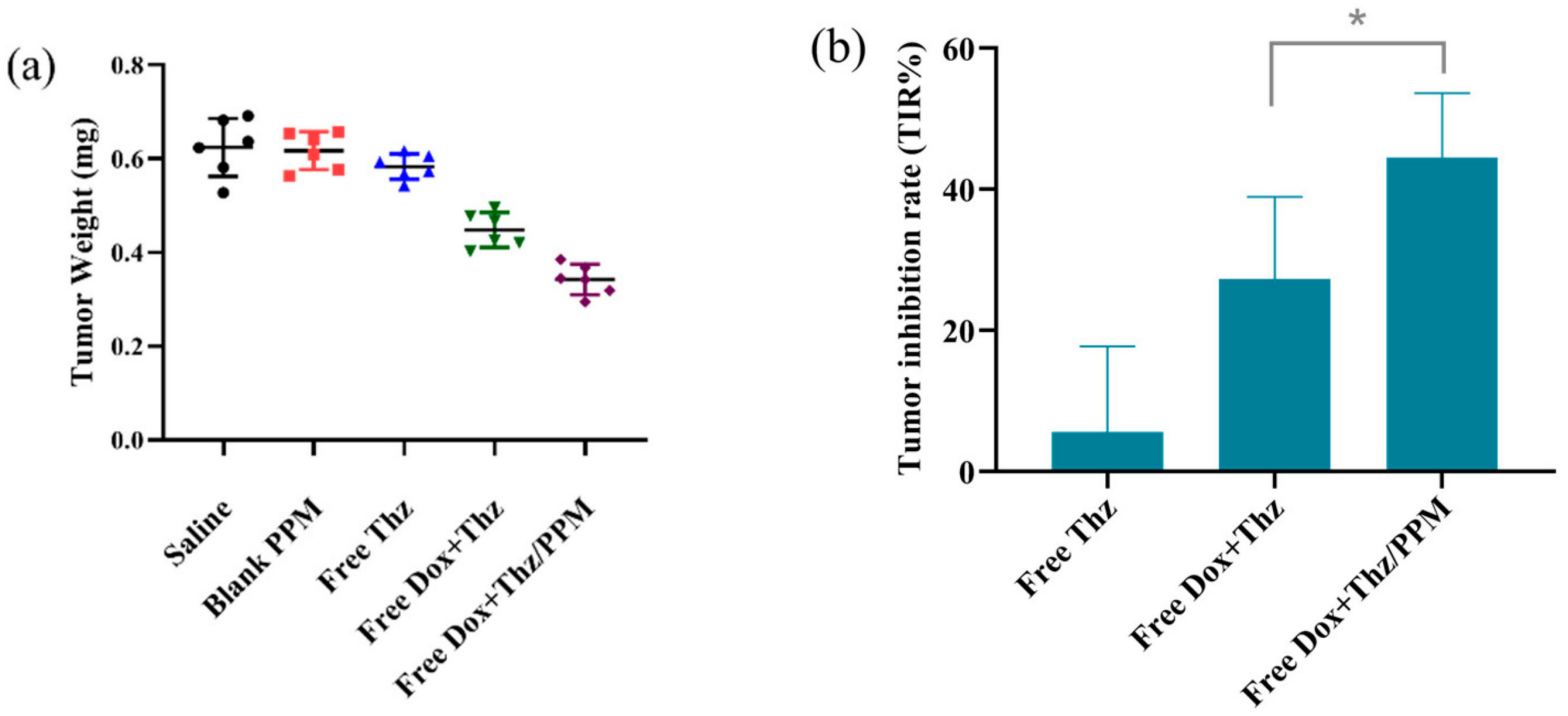
The tumor weight (**a**) and tumor inhibition rate (TIR%) (**b**) of xenograft tumor-bearing nude mice at the end of the test after intravenously-injected saline, blank PPM, Thz solution, Dox and Thz solution, and Dox solution and Thz/PPM (*n* = 6). * *p* < 0.05.

In vivo anti-tumor efficacy of different formulations in MCF-7 xenograft-bearing nude mice was investigated at the end of the test after 22 days of observation. As expected, blank PPM had no inhibitory effect on the tumor, and Thz solution did not show a significant anti-tumor effect because it only killed cancer stem cells. The combination of Dox and Thz solution had a certain anti-tumor effect but was incomplete, which could be attributed to their non-specific distribution in vivo. The synergistic Dox solution and Thz/PPM displayed significant tumor inhibition than Free Dox and Thz, which presents lower tumor weight (*p* < 0.05, Figure 10a,b). Better anti-tumor activity could be attributed to the micelles with a small size (<200 nm), which could be passively accumulated in the tumor due to the EPR effect. The in vivo anti-tumor study further showed that Thz/PPM, which targeted CSCs, improved the anti-tumor activity of Dox.

## 4. Conclusions

In this research, pH-sensitive PEG-PBAE block copolymers were successfully synthesized through a Michael-type step polymerization. Thz was successfully loaded into self-assembled PEG-PBAE micelles with a pH sensitive property. The Thz-loaded micelles with a round appearance and a particle size of about 100 nm have higher drug loading and entrapment efficiency. In a series of studies in vitro, the release of drug-loaded micelles, cytotoxicity, and cell uptake all showed pH-dependent behavior. In the lower pH environment, based on the protonation of PBAE, the Thz in drug-loaded micelles exhibited potential anti-CSCs cytotoxicity, effective internalization, and rapid drug release triggered by pH to achieve intracellular drug concentration. The cellular distribution indicated that the copolymer facilitated the effective endo-lysosomal escape of C6. The pH-sensitive micelles helped Dox to enhance anti-tumor efficacy. All the results demonstrated that the biocompatible, tumor-targeted, and pH-responsive Thz/PPM was an ideal drug delivery system to treat breast cancer by targeting breast CSCs.

## Figures and Tables

**Table 1:** Characterization results of PBAE and PEG-PBAE copolymers.

Copolymer	Mw(Da) ^a^	Mn(Da) ^a^	Mw/Mn ^a^	Mn(Da) ^b^	Mp	pKa
PBAE	5646	4773	1.18	5024	6129	6.6
PEG-PBAE	7325	6534	1.13	6915	8040	6.7

^a^ Determined from GPC analysis. ^b^ Calculated from ^1^H NMR spectrum.

## Supplementary Materials

The following are available online at https://www.mdpi.com/1999-4923/12/2/111/s1, Figure S1: FT-IR spectra of PBAE and PEG-PBAE. Figure S2: Acid–base titration profiles of PEG-PBAE copolymer with NaCl as control. Figure S3: Images of adherent MCF-7 cancer cells cultured in serum-containing medium (a) and mammospheres for BCSCs cultured in serum-free medium (b) under the light microscope (magnify 200 times). Figure S4: Identification of phenotype for the MS. (a. isotype control. b. stained with anti-CD44-FITC and anti-CD24-PE).

## Abbreviations

Thz, Thioridazine. CSCs, cancer stem cells. PPM, PEG-PBAE micelles. CMC, critical micelle concentrations. Thz/PPM, Thz-loaded PEG-PBAE micelle. MS, mammospheres. C6, Coumarin-6. EPR, enhanced permeability and retention. HDD, 1,6-Hexanediol diacrylate. TDP, 1,3-Bis(4-piperidyl) propane. MP, 4-Methylpiperidine. Human EGF, Human epidermal growth factor. FGF, Fibroblast growth factor. TMS, tetramethylsilane. CDCl_3_, chloroform-d. TEM, transmission electron microscopy. LC, loading content. EE, encapsulation efficiency. SDS, sodium dodecyl sulfonate. SD, standard deviation. ANOVA, analysis of variance. FLP, filipin. CC, colchicines. CPM, chlorpromazine. CLSM, Confocal Laser Scanning Microscope. TIR%, tumor inhibition rates. Dox, Doxorubicin.
