# Supplementary material for: Synthesis and Characterization of pH-Responsive PEG-Poly(β-Amino Ester) Block Copolymer Micelles as Drug Carriers to Eliminate Cancer Stem Cells
Source: Pharmaceutics. 2020 Jan 30;12(2):111. doi: 10.3390/pharmaceutics12020111 (PMC7076537; doi:10.3390/pharmaceutics12020111)
Supplement: Supplementary file 1 [file pharmaceutics-12-00111-s001.pdf]

pharmaceutics

Supplementary Materials: Synthesis and Characterization of pH-Responsive PEG-Poly(β-Amino Ester) Block Copolymer Micelles as Drug Carriers to Eliminate Cancer Stem Cells

Weinan Li, Jialin Sun, Xiaoyu Zhang, Li Jia, Mingxi Qiao, Xiuli Zhao, Haiyang Hu, Dawei Chen and Yanhong Wang

Results and Discussion

S1. FT-IR Characterization of PBAE and PEG-PBAE

Figure S1 showed the FT-IR spectra of PBAE and PEG-PBAE. In the spectra of PBAE, at wave number 1734 cm^{-1}, the new stretching vibration absorption peak of ester bond carbonyl group appeared, and methyl absorption peaks appeared at 2922.9 cm^{-1}, 2851.4 cm^{-1}, 1467.6 cm^{-1} and 1383.6 cm^{-1}, which indicated that PBAE was successful synthesized. In the spectra of PEG-PBAE, at wave number 1407.2 cm^{-1}, the absorption peak of olefin bond disappeared, indicating that the terminal olefin bond of PBAE was linked to the amino group on PEG, which indicated that the synthesis of PEG-PBAE was successful.

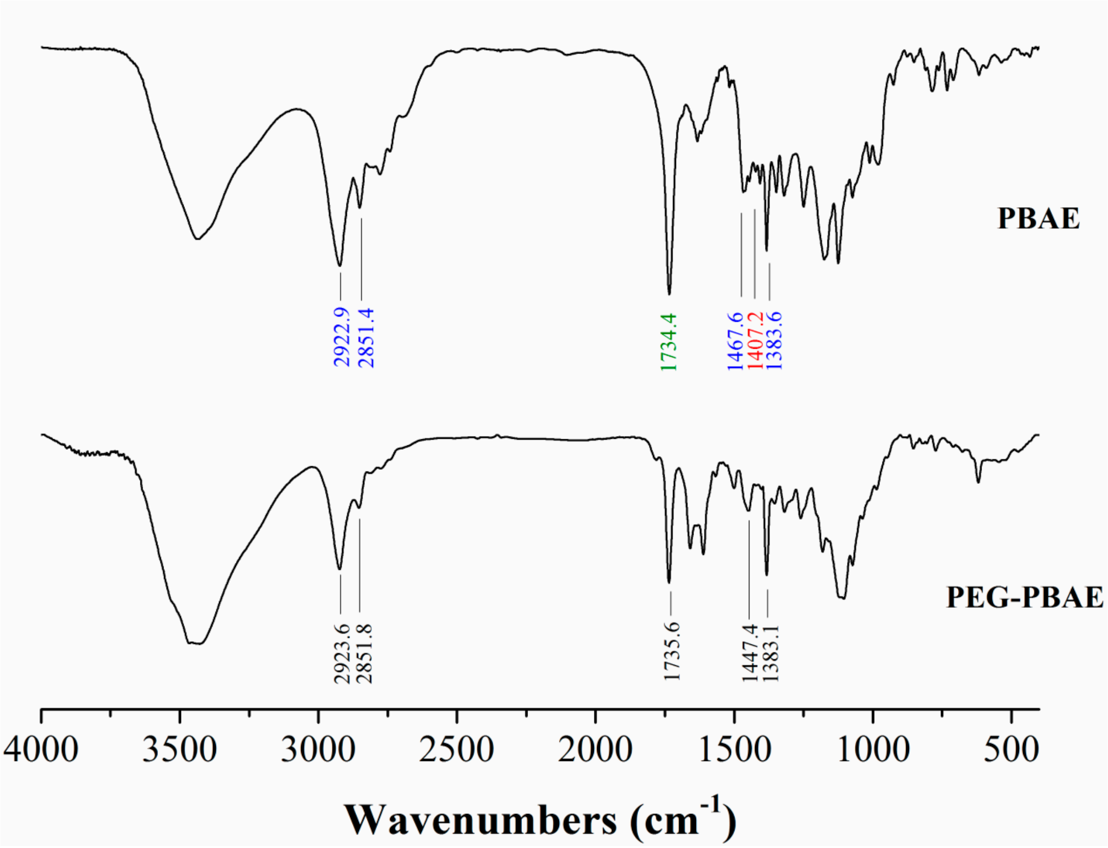

Figure S1. FT-IR spectra of PBAE and PEG-PBAE.

S2. pH Sensitivity of Copolymers

The pKa of the copolymers was studied by acid–base titration. The results of acid-base titration of polymers were shown in Figure S2. In the titration process, the pH value of control (NaCl solution) increased rapidly to 11, and there was no plateau period. However, PEG-PBAE polymer showed strong buffer capacity in the pH range from pH 4.9 and 8.5, indicating that PBAE block in

the copolymers was accounted for in the buffering capacity. Based on the *Henderson-Hasselbalch* equation, the pKa was about 6.7.

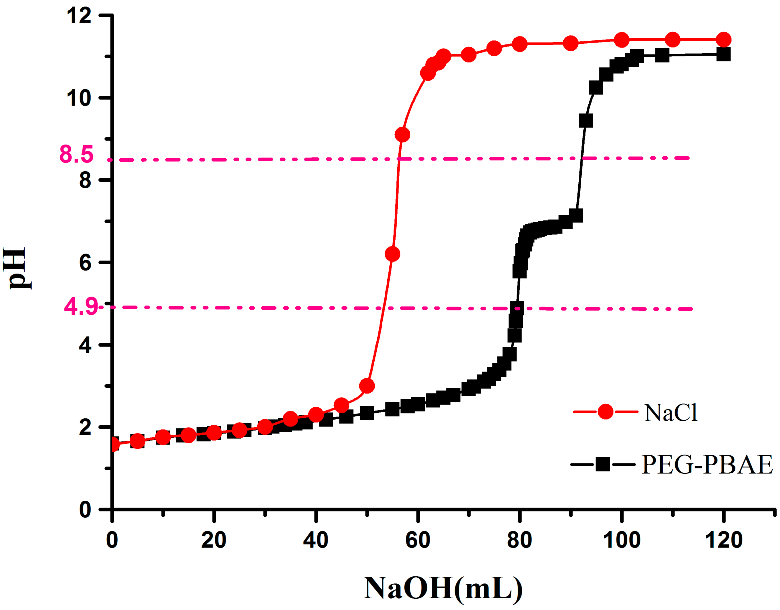

Figure S2. Acid–base titration profiles of PEG-PBAE copolymer with NaCl as control.

S3. Sorting and Identification of Breast CSCs

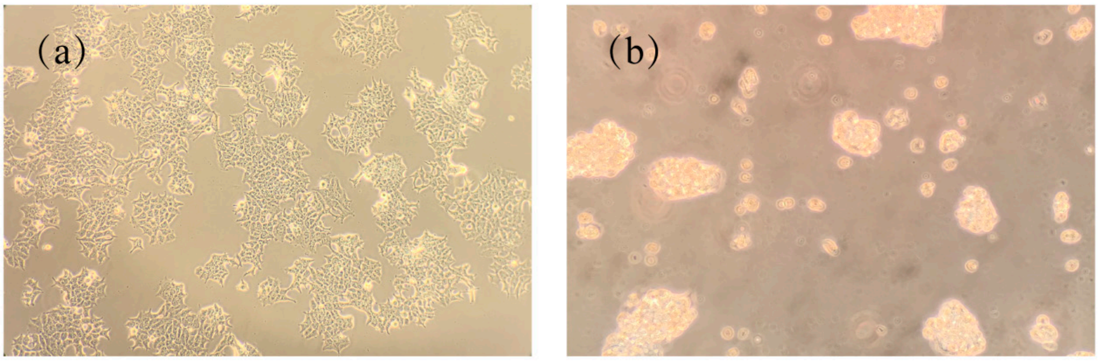

Figure S3. Images of adherent MCF-7 cancer cells cultured in serum-containing medium (**a**) and mammospheres for BCSCs cultured in serum-free medium (**b**) under the light microscope (magnify 200 times).

As shown in Figure S3. MCF-7 cells were incubated in serum-containing DMEM medium , and adhered to the bottle wall (As shown in Figure S3a). After adherence, the cells were digested by trypsin and incubated in serum-free DMEM-F12 medium at a certain density. MS could be observed several days later (Figure S3b).

Anti-CD44-FITC and anti-CD24-PE were used to identify the surface antigens of MS cells incubated in serum-free medium. As shown in Figure 4, compared with the control group (a), the results showed that MS cells exhibited the phenotypic characteristics of $CD44^{+}/CD24^{-}$ and the content of MS cells (b) was 95.4%, which was consistent with the phenotypic characteristics of breast cancer stem cells reported in literature [1].

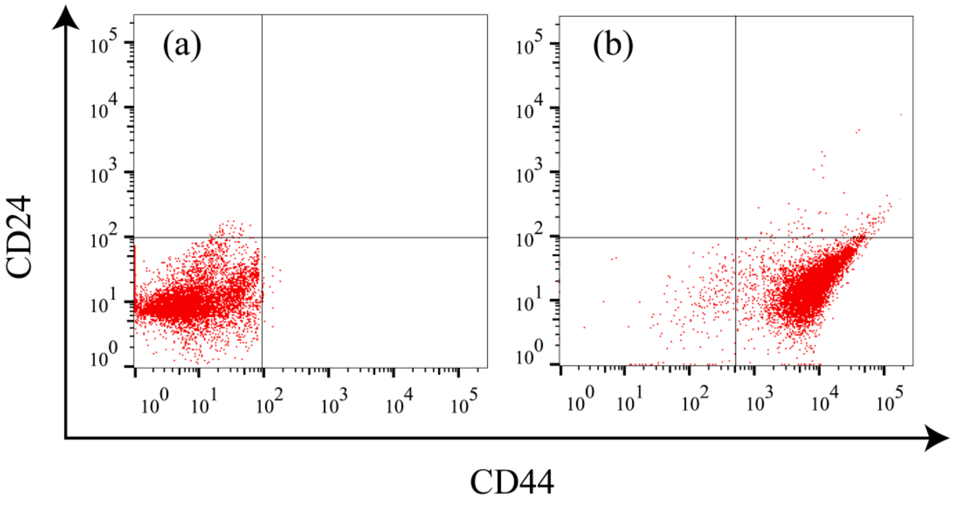

Figure S4. Identification of phenotype for the MS. (**a**. isotype control. **b**. stained with anti-CD44-FITC and anti-CD24-PE).

[1] Zhang, L.; Yao, H.J.; Yu, Y.; Zhang, Y.; Li, R.J.; Ju, R.J.; Wang, X.X.; Sun, M.G.; Shi, J.F.; Lu, W.L. Mitochondrial targeting liposomes incorporating daunorubicin and quinacrine for treatment of relapsed breast cancer arising from cancer stem cells. *Biomaterials.* 2012, 33, 565–82.
